# Effect of Serial Passage on the Pathogenicity and Immunogenicity of Vaccinia Virus LC16m8 Strain

**DOI:** 10.3390/biology10111158

**Published:** 2021-11-09

**Authors:** Akiko Eto, Norio Yamamoto, Yasuhiro Kanatani

**Affiliations:** 1 Department of Health Crisis Management, National Institute of Public Health, 2-3-6 Minami Wako, Saitama 351-0193, Japan; eto.a.aa@niph.go.jp; 2 Department of Virology, School of Medicine, Tokai University, 143 Shimokasuya, Isehara 259-1193, Japan; n-yamamoto@tsc.u-tokai.ac.jp; 3 Department of Clinical Pharmacology, School of Medicine, Tokai University, 143 Shimokasuya, Isehara 259-1193, Japan

**Keywords:** vaccinia virus, vaccine, LC16m8, immune response, humoral immunity, interindividual difference

## Abstract

**Simple Summary:**

There is great current interest in vaccines for use in the coronavirus disease 2019 (COVID-19) pandemic. Safety and efficacy are particularly important in vaccine development, and these depend on the properties of the vaccine and the response in the vaccinated person. Some vaccines produce a strong immune response but may also cause adverse events in certain individuals. It is particularly important to predict if a person may be vulnerable to these adverse events, and this is one of the purposes of the field of vaccinomics. In this review, we examine recent studies on vaccinia virus (VACV) vaccine, which is the key vaccine responsible for eradicating smallpox. In particular, we consider the different individual responses to this vaccine, and we discuss future research directions in this area.

**Abstract:**

The phenotype of an attenuated live vaccine depends on gene mutation achieved by, for example, many passages in cultured cells. Viral clones with preferable phenotypes are selected and the causative genetic mutation(s) are later identified. LC16m8 is an example of a highly attenuated smallpox vaccine that was developed and licensed in Japan in the 1970s. LC16m8 was obtained by the passaging of Lister strain, with indicators of small plaque formation and temperature sensitivity as virus phenotypes. This strain can replicate in mammalian cells and provides robust cellular and humoral immunity, as well as long-term immune memory. Recent studies using proteome-wide antigen arrays have revealed that antibody production against LC16m8 and other VACVs differs largely among individuals. Moreover, associations between SNPs in immune-related genes and immune outcomes have been increasingly found. These results lead to predicting adverse events of a vaccine, which is a purpose of vaccinomics. Studies on VACV will continue to contribute to the understanding of host-pathogen interactions and to development of a vaccine for other infectious and non-infectious diseases. Here, we review studies of VACV, including our recent research on LC16m8, with a focus on the phenotype and genotype, and we discuss future research directions.

## 1. Introduction

The development of vaccines is currently underway on a global scale, as measures against the coronavirus disease 2019 (COVID-19) pandemic. In vaccine development, high degrees of safety and efficacy are required, and these depend on multiple factors on the vaccine side and the host side, including human leukocyte antigen (HLA) gene polymorphism [1,2]. Live vaccines can induce robust humoral and cell-mediated immunity in the host, but may cause adverse events, some of which are serious. Therefore, ensuring safety while retaining immunogenicity is a major challenge in live vaccine development. Live vaccines can be attenuated by passaging during cell culture [3]. When grown under unfavorable conditions, such as in animals or cell types other than naive (natural) hosts, viruses exhibit novel phenotypes resulting from gene mutation(s) that occur in passaging, and some of these phenotypes show low virulence in humans. Thus, passaging provides an empirical method for obtaining attenuated viral clones with preferable phenotypes, and the causative genetic mutation(s) can then be identified by further characterization.

The VACV vaccine has greatly contributed to the eradication of smallpox and thus, is considered the most successful vaccine to date [3]. LC16m8 is an attenuated VACV developed from the Lister strain by passaging in cell culture, and has been used as a VACV vaccine [4,5]. In the course of passaging, mutations in specific region related to the biological activity (plaque size and pock size; the ability of the virus to disseminate from cell to cell) of VACV in host cells were suggested to be involved in the weakening of LC16m8 [6,7]. Here, we describe the phenotypes and genotypes of LC16m8 with respect to their efficacy and safety, recent advances in the understanding of the biological mechanism related to these phenotypes and genotypes, and the potential use of LC16m8 for preventive and therapeutic purposes.

## 2. Safety of VACV Vaccine through the LC16m8 Phenotype

### 2.1. Development of the Attenuated VACV Strain LC16m8

The concept of preventive inoculation of a cross-immunizing but clinically mild poxvirus to prevent severe cases of smallpox was established by Edward Jenner in the 18th century, and routine vaccination against other microorganisms is nowadays widely accepted in most countries. In the 1950–1970s, the World Health Organization (WHO) developed the global smallpox eradication program, in which first-generation smallpox vaccines were used. These vaccines were fabricated in calf skins and caused rare but serious adverse events, particularly encephalitis among infants [8]. As the number of patients infected with smallpox decreased, this concern became greater with regard to a risk-benefit assessment, and more efforts were made to develop safer vaccine strains. To establish attenuated VACV strains, Hashizume et al. [4] cultured the VACV Lister strain for numerous passages in primary rabbit kidney cells at 30 °C to select for temperature-sensitive clones. Clones were selected based on their ability to form small pocks on the chorioallantoic membrane of embryonated eggs.

For safety profile analysis, the newly established virus clone LC16m8 was compared with the parental Lister and intermediate LC16m0 strains in animal models. This research focused on neurovirulence and local reactions, and included experiments in which viruses were inoculated intracerebrally into rabbits and monkeys, intraperitoneally into mice to investigate spreading into the central nervous system (CNS) via the blood stream, and intradermally into rabbits to examine reactions at the inoculation site [4]. In rabbits and monkeys, LC16m8 was not detected in the brain, in contrast to the other virus strains. In mice, LC16m8 was also found not to transfer into the brain, although it was present in the blood for several days post-inoculation. Overall, these tests showed that LC16m8 had a much safer profile (small plaque formation and weaker dermal reaction) compared to the parental Lister and intermediate LC16m0 strains. Consequently, administration of LC16m8 to children resulted in a reduced frequency of fever, and no serious adverse events [4].

Subsequent genetic studies revealed that these properties of LC16m8 were due to a point mutation in the B5R gene [6,7]. B5 is a 42-kDa glycosylated type I membrane protein found in the extracellular enveloped virion (EEV, also called EV) membrane [9,10]. The protein contains a 19mer signal sequence, 4 short consensus repeats (SCRs), a 51mer stalk domain, a transmembrane domain, and an intracellular domain [7,9,10]. B5 localizes to the EEV membrane with a large ectodomain of 275 amino acids [9,10]. The sequences of B5 proteins are highly conserved among VACV strains [10,11].

### 2.2. Functions of B5 Protein and Extracellular Enveloped Virion, and Adverse Events of the Vaccine

**Figure 1:**
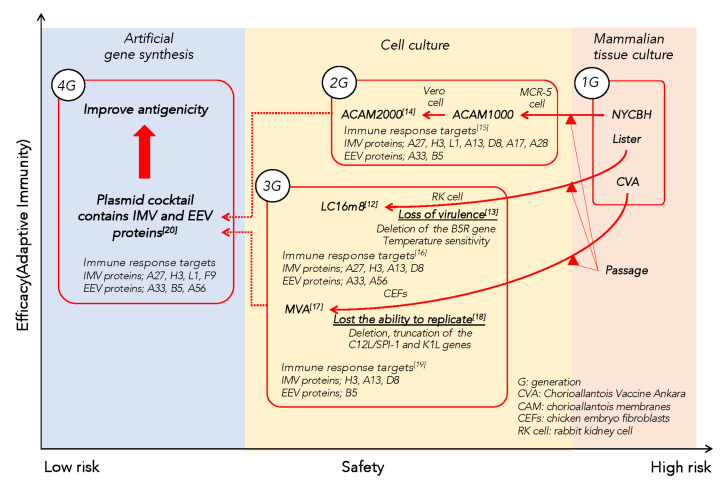
Classification of smallpox vaccine based on safety and efficacy. The different smallpox vaccines can be classified into 1st to 4th generations that are positioned as shown in the figure from the viewpoint of safety and efficacy. Safety is the avoidance of the risk of adverse events, and efficacy can be assessed by adaptive immunity, including cell-mediated immunity (take rate) and antigenicity. The first-generation NYCBH, Lister and CVA strains, which are considered to be the parent strains, were switched in cell culture to produce second-generation ACAM1000 and ACAM2000 with improved quality and efficiency in mammalian cell culture. Cell culture passaging shifts to the third generation LC16m8, which has weakened infectivity, and MVA, which has a weakened ability to replicate. IMV proteins, A27, H3, L1, A13, D8, A17, A28, and EEV proteins B5 and A33 are neutralizing antigens. The 4th generation is obtained by the artificial synthesis of highly immunogenic EEV and IMV antigens, based on findings obtained during the development of the 1st to 3rd generations [20].

In LC16m8, the point mutation in the B5R sequence causes truncation to a polypeptide, containing only amino acids 1–92 of the complete protein [7,11]. Due to this mutation, the B5 protein of LC16m8 lacks its proper localization, function, and antigenicity. The safety of LC16m8 depends on this mutation in B5. VACVs have improved from the first to the fourth generation with distinct safety and efficacy profiles (Figure 1) [5,12,13,14,15,16,17,18,19,20]. B5 is involved in formation of EEV, which is formed from another infectious virion, intracellular mature virion (IMV, also called MV) via two precursor forms, intracellular enveloped virion (IEV), and cell-associated enveloped virion (CEV), through intracellular transport, the addition of membrane (wrapping) and exocytosis. IMV and EEV are two major infectious virions. Production of EEV is only about 1% that of IMV, with differences depending on the VACV strain, but EEV is largely responsible for the virulence of the virus [21].

The properties of EEV of rapid spreading, dissemination between organs and immune evasion are all related to the enhancement of virus virulence [22,23]. Regarding immune evasion, EEV possesses fewer neutralizing membrane antigens than IMV, which has multiple neutralizing antigens. EEV also integrates membrane proteins derived from host cells, which enables the virion to escape the host immune system [22]. EEVs infect cell-to-cell via superinfection exclusion, which is referred to as “superinfection repulsion” [24]. Cells infected by VACV express surface proteins as a mark of infection. Viruses can then infect uninfected cells selectively, which results in a faster spread than the estimated time based on the replication kinetics [24].

VACV has a tropism for skin, and the virus replicates locally at the vaccination site, and then in lymph nodes and the spleen. Other target tissues of the virus include the kidney, heart, and nervous system. Thus, these tissues are related to adverse events, particularly in immunosuppressed individuals. As noted above, post-vaccination encephalitis among infants was a particular concern until the 1970s [8]. The neurovirulence of VACV requires transmission of the virus from the bloodstream to the brain, and its replication in the brain. EEV is responsible for dissemination between organs and tissues in the host. The neurovirulent VACV passes through the blood–brain barrier in migrating from the blood to the brain [25], and the growth of the virus in the brain has been assessed in a mouse model [26]. Viruses are neurovirulent and cause neuropathogenicity. Neurotoxicity is limited to chemical compounds and the bacteria that can produce neurotoxic compounds [27]. Therefore, an evaluation system using suckling mice was developed. 

B5 is a dominant antigen in antibody production and T cell epitopes. Class I and class II epitopes [28,29], as well as several B cell epitopes [30], have been identified. The T-cell epitopes of VACV B5 protein registered in the International Epitope Database (IEDB) [28,29,31,32] are shown in Table 1. These epitopes reside between amino acids 1–92, which are included in LC16m8 B5 protein, and 93–317, which are lost from a truncated form of LC16m8 (Table 1), based on 2 studies [28,29], in which peptides derived from B5 and selected proteins were tested as candidates for T cell epitopes. In MHC class I-deficient mice, protection against lethal challenge of virulent vaccinia WR (Western reserve) strain is provided by LC16m8, Lister, and inactivated Lister strain, suggesting a contribution of immune mechanism independent of MHC class I [33]. The relationship of loss of immunogenicity of B5 to improved safety of LC16m8 via cell-mediated immunity [34] still remains to be studied. 

### 2.3. Genotype-Phenotype Factors Related to Adverse Events after Vaccination

We studied factors related to adverse events following the vaccination of LC16m8 in primary and re-vaccination groups [35]. The frequency of adverse events was analyzed with detailed examinations of skin reactions at 10–14 days post-vaccination, background data, blood pressure, and clinical laboratory tests. Among 268 individuals, no serious adverse events were reported, and there were more mild adverse events among primary vaccinees than re-vaccinees. There were tendencies that rates of adverse events were increased with a larger size of erythema or blister. 

Cytokine levels in host cells have been reported to be associated with adverse events caused by VACV [36]. The innate immune system recognizes invading pathogens through pattern-recognition receptors and transmits signals to initiate humoral and cellular immunity. Cytokines and their receptors bridge between innate and adaptive immunity, and thus, polymorphisms in these molecules are likely to influence immune response [2]. Compared to wild type mice, IL-1R knockout mice have a larger local reaction after scarification with VACV, which indicates virus replication at the inoculation site. These mice also develop eczema vaccinatum, which is similar to atopic dermatitis, a serious adverse event in humans. These phenomena occur without an influence of systemic immunity, indicating the critical role of IL-1R in the local inflammatory response against VACV [37].

Studies have been conducted to identify SNPs that are significantly associated with levels of cytokine secretion. Among candidate cytokine/cytokine receptor genes, two SNPs were identified in Caucasian and African American cohorts: an IL1RN (Interleukin 1 Receptor Antagonist) gene regulatory SNP (rs452204) associated with IL-2, and an IL12RB2 gene SNP (rs3790567) associated with IL-1β secretion [38]. SNPs in several genes associated with immune function, signal transduction, and other functions have also been linked to cytokine levels by GWAS (Genome-Wide Association Study) [39]. Stanley et al. [40] investigated the relationship of fever after smallpox vaccination with SNPs in 19 candidate genes, and found an association between fever and certain haplotypes in the IL-1 gene complex and in IL-18 and IL-4. Reif et al. Ref. [41] similarly investigated links of adverse events after smallpox vaccination with 36 SNPs in 26 candidate genes, and found an association of fever with MTHFR (methylenetetrahydrofolate reductase) and IRF(interferon regulatory transcription factor)-1 genes in two independent studies. MTHFR has also been reported to have an association with adverse events of drugs [41]. These findings led to the development of a model based on genetic data and proteomic (cytokine production) data, which included ICAM-1, IL-10, G-CSF and IL-4 [42].

## 3. Efficacy of VACV Vaccine through a Personalized Public Health Focus

### 3.1. Immune Protection against VACV by Antibodies

Neutralizing antibodies play a pivotal role in protection against orthopox virus [43]. Although the correlation with protection is unclear, possessing antibodies beyond a certain level is sufficient for protection. After recovery from smallpox, immunity usually lasted lifelong and re-infections were barely ever reported. The VACV vaccine also provides protection for decades, particularly for humoral immunity. Administration of vaccinia immune globulin (VIG) is efficient against vaccinia infection, and VIG is used for preventive and therapeutic purposes. VIG contains multiple specific antibodies, including neutralizing antigens H3, A27, and WR148 of IMV, and A33, A56, and B5 of EEV [44]. Neutralizing monoclonal antibodies are promising candidates as novel vaccine or therapeutic agents [45]. Protection following administration of a single antigen has been reported, with the targets of neutralizing antigens including A17, A27, A28, D8, H3, and L1 for IMV, and B5 for EEV (Figure 1). The combination of antibodies against different antigens against both IMVs and EEVs particularly increases protection [45,46].

In the post-genomic era, proteome-wide approaches can be used to evaluate whole pathogen antigens. In 2005, Davies et al. [47] reported a protein array platform with quasi-whole proteome antigens for the comprehensive examination of antibody production against VACV. Analysis of the VIG identified 14 antigens, including D13, D8, L4, A10, and H3 [47]. Probing of sera from vaccinated macaques and mice revealed that antibody profiles were overall similar among three species, with eleven common antigens identified [47]. Five archival sera samples from convalescent smallpox patients from Africa and Indonesia [48] included overlap antibodies with Dryvax-vaccinated sera, demonstrating cross-reactivity on a molecular level between smallpox and VACV. Analysis of sera from patients who received Dryvax [48,49,50] showed that antibody production varies enormously among individuals in both specificity and quantity, showing that the specific antibody produced after vaccination differs on an individual basis. 

An array comprised of an E.coli cell-free system [47] may result in the structure or modification of antigens differing from those produced in mammalian hosts. Other groups have utilized antigen arrays with eucaryotic expression systems, such as Sf9 cells [51]. The antibodies identified in these systems were generally similar to those previously reported. Examinations of antibody production in various animal models, including mice, rabbits, monkey and prairie dog, have shown similar antibody profiles among species. A comparison of antibody production in humans by NYCBH [15,19,48,49,50,51,52,53,54,55,56], ACAM2000 [15,56], MVA [19,54], and LC16m8 [16] has shown similar antigenicity among these strains, reflecting the high sequence homology among the virus strains.

### 3.2. Antibody Profile Induced by LC16m8

After the bioterrorism events in 2001 in the United States of America, the need for a smallpox vaccine was reconsidered in several countries. In Japan, LC16m8 was reproduced and stockpiled, and their immunogenicity and frequency of adverse events were evaluated [34,35,57]. In 2002–2005, a clinical study of the safety and efficacy of LC16m8 was conducted in >3000 adults, including both primary vaccinees and revaccinees. In participants, neutralizing antibody response and local reactions, which are two indicators for the success of smallpox vaccination, were highly elicited [57]. Cellular immunity was also as compatible as the first-generation vaccine, Dryvax, in a comparative study [34]. LC16m8 could boost pre-existing immunity [35,57], as well as provide long-term immunity [35].

We have investigated the antibody profile induced by LC16m8 vaccination [16]. In Japan, until the 1970s, three VACV strains were approved for routine vaccination, and vaccinees were categorized into four groups according to the number of previous vaccinations (0–3 times) [57]. Paired sera from 200 individuals were examined to evaluate antibody production against LC16m8 by protein microarray [16]. Importantly, the antibody profile in pre-vaccination sera of re-vaccinees represents the residual immunity conferred by routine immunization performed during childhood. Antibodies against various antigens, including neutralizing antigens such as H3 and A27, were found to have remained for decades. Upon vaccination with LC16m8, both primary vaccinees and re-vaccinees yielded antibodies against multiple antigens, including antibodies for both IMV and EEV. In primary vaccinees, LC16m8 failed to induce EEV-neutralizing antibody and, consistent with this observation, antibodies to EEV protein B5 were not produced; however, a boosting response against B5 protein has been observed in re-vaccinees [58]. The antibody profile induced by LC16m8 was generally similar to those previously reported, indicating that the immunogenicity of LC16m8 is comparable to those of first-generation vaccines. Our results for LC16m8 vaccination provide important findings, since they were obtained in a relatively large number of individuals with a clear vaccination history and limited unknown exposure after discontinuation of routine vaccination. Thus, these results contribute to the understanding of long-term immunity against VACV [35].

### 3.3. Genotype-Phenotype Status Related to Antibody Production in Response to VACV Vaccine

Genetic factors associated with antibody production have been investigated for HLA, cytokines, and receptors, or in GWAS [59,60,61]. HLA class II DQB1*03:02 has been reproducibly shown to be associated with high neutralizing antibody titers in two cohorts vaccinated with ACAM2000 [59]. Among 32 cytokine and cytokine receptor genes, haplotypes in the IL18R1 (interleukin-18 receptor 1) gene were associated with neutralizing antibody titers in Caucasians and African Americans [60]. In the same cohort, GWAS was conducted to identify SNPs that were differentially associated with antibody production in Caucasians, African Americans, and Hispanics, with the goal of finding genes that may be involved in the regulation of humoral immunity [61]. For the association with IFNγ levels, GWAS revealed multiple SNPs in genes that directly regulate IFNγ-mediated T cell functions [39]. In addition to genetic factors, gender effects are important in smallpox vaccination, with females showing more robust antibody production [62]. 

## 4. Future Research Directions

### 4.1. Vectors for Infectious and Non-Infectious Diseases 

The use of VACV as a vaccine vector for other infectious diseases or cancers has been investigated since the 1980s [63,64,65], due to its many desirable characteristics as a vector [66,67]. The stability of VACV (i.e., resistance to environmental changes, including temperature, moisture, and ultra-violet rays) and ease of administration (bifurcated needles, and a local reaction as an indicator of vaccination success) contributed largely to the success of smallpox vaccination and eradication. VACV elicits robust cellular and humoral immune responses and long-term immune memory, and has been used for an extended period of time in humans as a smallpox vaccine. It has a broad host range beyond humans and broad tropism in terms of cell type [66,67]. There are several safety advantages to using VACV as vectors. Firstly, exogenous genes are not integrated into the host nuclear genome because VACV replication occurs in the cytoplasm. Secondly, low mutation rate, and deletion of several genes responsible for immune evasion may improve its safety and immunogenicity (Figure 1). Thirdly, practical advantages such as lyophilization procedures have been well established. The large genome of VACV enables the insertion of more than 10 kb of foreign DNA for expression as immune antigens. Applications include antigen expression of infectious agents for human and animal diseases, immunotherapeutic cancer vectors, and oncolytic cancer therapy vectors [67]. In addition, the EEV plays an important role in virus virulence, and thus, the absence of EEV seems preferable for a vaccine vector with regard to safety [6]. Relatively few studies of LC16m8 for vector development have been performed, but it is a promising backbone based on its properties and current findings. Studies on LC16m8 as a vector backbone have been reviewed by Sugimoto [68], Kidokoro [69], and Yoshikawa [70]. 

### 4.2. Development of Next-Generation Vaccines Based on Interindividual Differences

The characteristics of VACV vaccine strains are shown in Table 2. ACAM2000 is a derivative of NYCBH developed in cultured cells, with the aim of establishing a single-clone vaccine strain with similar efficacy to that of the first-generation Dryvax strain. Dryvax is a multi-clone vaccine, and thus, can manifest a significant pathogenicity. ACAM2000 possesses a similar efficacy to Dryvax, as well as a similar pathogenicity [71]. The CDC describes contraindications to smallpox vaccine, based on: (1) underlying immunosuppression or treatment with immunosuppressive drugs, (2) atopic dermatitis or other skin problems, (3) pregnancy, and (4) newborn infants (age < 1 year) [72]. ACAM2000 has been recommended as a stockpile vaccine strain, together with LC16m8, by a WHO Expert Committee, which considered the importance of a visible sign of local reaction for ease of the evaluation of vaccination success [73].

LC16m8 possesses a balanced safety and efficacy profile, with no severe adverse events, such as cardiac diseases or encephalitis. Since LC16m8 was isolated as a temperature-sensitive strain, it cannot replicate in the brain, in which the temperature is higher than in other tissues [6]. LC16m8 has been licensed for children and adults, without age restriction [35]. A more moderate restriction is used for vaccination of individuals with skin diseases [57]. 

The MVA strain lacks a large portion of the genome, and this prevents replication in mammalian cells [17,18]. Since MVA is administered intramuscularly or subcutaneously, it is not possible to compare the take rates observed with other VACV vaccine strains. However, in a phase III trial of adults aged 18 to 40 years, strong local reactions were observed with 60% erythema, 50% swelling, and 45% induration after subcutaneous inoculation. Moreover, there were no serious complications such as myocarditis or pericarditis, and the seroconversion rate was shown to be extremely high at 99.8% [75]. As shown in Figure 1, the profile of antibodies induced after MVA inoculation includes H3, A13, D8, and L1 as IMV and B5 as EEV. This profile is similar to that of antibodies induced after Dryvax inoculation [19]. Recently, MVA have been shown to enhance cellular immunity by activating IRF-3 via the cGAS (cyclic GMP-AMP synthase)—STING (stimulator of interferon genes) pathway [76]. The immunogenicity and immunomodulatory effects of MVA are comparable to that of the first-generation VACV vaccines, despite the deficiency for replication in most mammalian cells. Therefore, MVA has been considered to be a safer candidate for the population that is immune-compromised by AIDS, chemotherapy for cancer, or immunosuppression therapy.

In the VACV vaccine, the transition from the first to second-generation smallpox vaccine included the improvement of the sterilization of the vaccine during the manufacturing process, since the first-generation vaccine was produced by inoculating the virus in calf skin [3]. Production efficacy was also improved by using cell culture, leading to phenotype manipulation during passaging. Since immunogenicity and pathogenicity appear to be double-sided, a large modification in antigenicity may lead to a less effective vaccine in the development of third-generation vaccines. Third-generation LC16m8 maintains its ability to replicate in mammalian cells, and induces both humoral and cellular immunity in the host immune system. The success of various live attenuated vaccines suggests the importance of biological and immunological characterization of factors on the host-side and vaccine-side. A serious adverse event is rare in vaccination, which makes the detection of rare adverse events difficult in clinical studies preceding mass vaccination. However, since immunogenicity and pathogenicity are successive phenomena derived from an immunological mechanism that should provide a deeper understanding of efficacy and an understanding and prediction of safety and pathological mechanism. Such prediction will allow the identification of individuals who should and should not receive a vaccine.

The transition from third- to fourth-generation vaccines is based on findings related to immune responses, including highly reactive epitopes. Specific antigens are selected after understanding the mechanisms underlying the relationships between the host and pathogen. These vaccine components can be manufactured in vitro.

Evaluation of antibody production should include both the quantity and function of neutralizing antibodies. Antibodies do not always function in a host-preferable manner, but may manifest pathologic effects, as seen in the phenomenon of antibody-dependent enhancement. Past exposures to pathogens, including unrelated pathogens, may affect vaccination outcomes through innate trained immunity, or cross-reactivity in adaptive immunity. All of these factors contribute to inter-individual differences. The VACV is an excellent system for studying immune response. Since natural exposures are largely unknown and difficult to control, the effect of existing immunity is one of the difficulties in studying the mechanism of the immune response to vaccines. In this respect, natural exposure to VACVs is limited after the eradication of smallpox and discontinuation of regular vaccination of VACV vaccine. Thus, the immune response elicited solely by vaccination can be evaluated in humans, as shown in our studies [16]. VACV induces a robust, innate and acquired immunity, which makes the virus an excellent system to elucidate the mechanism of trained immunity. Therefore, biological information obtained from VACV studies continues to contribute to future vaccine development. In addition, LC16m8 is a well-tolerated strain, and immunoglobulins mimicking the antibody repertoire induced by LC16m8 are promising therapeutic agents. As shown in Figure 1, studies of the VACV have suggested the significance of A27, H3, and L1 as IMV proteins, and A33 and B5 as EEV proteins.

## 5. Conclusions

Advanced understanding of immunological characteristics of the immune response to vaccination has enabled development of vaccines based on specific and highly reactive epitopes. However, methodology for the evaluation of safety and efficacy of vaccines has largely remained unchanged, and needs to be modified based on differences in the immune response. Nevertheless, omics data including multi-level data for genetic polymorphism, mRNA expression, and protein expression are large and difficult to analyze in an integrated manner, and manipulation of these data to extract necessary information is a major technical challenge. Databases for integrated analysis are required, and open data are essential to assure research transparency, but, at the same time, these data include highly private and sensitive personal information, that must be protected. These challenges require extensive discussion beyond the science community.

## Figures and Tables

**Table 1:** T-cell epitopes in B5 protein.

T–Cell Epitopes of Vaccinia Virus B5 Protein	First–Last Amino Acid	Epitope Sequence	HLA Class														Ref.
			Class I				Class II										
			A 2	B 35	B 44	B 55	DR1	DR3	DR4	DR7	DR 11	DR 13	DR 15	DR B3	DR B4	DR B5	
T–cell epitopes that reside in B5 protein of LC16m8	1–15	MKTISVVTLLCVLPA					*	*	*	*	*	*	*	*	*		[28]
	4–18	ISVVTLLCVLPAVVY					*	*	*		*	*					[28]
	5–19	SVVTLLCVLPAVVYS	*														[29]
	79–93	SDYISELYNKPLYEV											*				[28]
T–cell epitopes of B5 protein that are lost from a truncated form of LC16m8	105–119	TKYFRCEEKNGNTSW			*												[29]
	166–180	ASYISCTANSWNVIP					*		*	*		*	*	*	*		[28]
	174–188	NSWNVIPSCQQKCDI							*		*						[28]
	193–207	NGLISGSTFSIGGVI				*											[29]
	204–218	GGVIHLSCKSGFILT					*	*	*	*	*	*	*		*	*	[28]
	225–239	CIDGKWNPILPTCVR		*													[29]
	269–283	QEIESLEATYHIIIV						*		*			*				[28]

* Human T cell epitopes in B5 protein of VACV registered in the Immune Epitope Database (IEDB). Refs. [31,32] were shown.

**Table 2:** Characteristics of VACV vaccines of the first, second, and third generations.

Vaccinia Virus Vaccines	Effectiveness		Safety	Vaccine Characteristics			Host Factors			
	Cellular Immunity	Humoral Immunity								
	Take Rate	Sero- Conversion	Adverse Events	B5R	Temperature Sensitivity	Plaque Formation	HLA	Genetic Polymorphism	Comorbidities	Ref.
Lister	High	High	Temporary abnormal electro- encephalogram	Yes	No	Large	Not found	Not found	Usual contraindications to vaccination	[4,7,27,71],
NYCBH	High	High	Myo-/ pericarditis	Yes	No	Large	Neutralizing antibody titers	Neutralizing antibody titers, IFNγ secretion, cytokine production, adverse events (fever)	Usual contraindications to vaccination	[14,38,39,40,41,59,60,61,62,70,71]
ACAM2000	High	High	Myo-/ pericarditis	Yes	No	Large	Neutralizing antibody titers	Not found	Usual contraindications to vaccination	[14,59,70,71]
MVA	ND	High	No cardiac events	Yes	No	No	Not found	Not found	Less restriction for immunodeficiencies, immunosuppressive therapies or atopic dermatitis	[17,72,74,75]
LC16m8	High	High	No cardiac events	No	Yes	Small	ND	ND	Less restriction for atopic dermatitis if skin lesions are dry and stable	[4,7,27,57]

## Data Availability

Not applicable.
